# A Prospective Clinical and Radiological Study of Symptomatic Upper Lumbar Disc Herniation in the Indian Population

**DOI:** 10.7759/cureus.102321

**Published:** 2026-01-26

**Authors:** Shiv Kumar Bali, Sandesh Subhash Agrawal, Bharat R Dave, Mikeson Panthackel, Ajay Krishnan, Shivanand C Mayi, Ravi Ranjan Rai, Mirant B Dave, Mahesh Sagar, Amritesh Singh

**Affiliations:** 1 Orthopedics, Spine Surgery, Stavya Spine Hospital and Research Institute, Ahmedabad, IND; 2 Orthopaedics, Sri Devaraj Urs Medical College-Sri Devaraj Urs Academy of Higher Education and Research Centre (SDUMC-SDUAHER), Kolar, IND; 3 Orthopaedics and Traumatology, Shri Balaji Institute of Medical Sciences (SBIMS), Raipur, IND; 4 Spine Surgery, Stavya Spine Hospital and Research Institute, Ahmedabad, IND; 5 Spine Surgery, Shree Narayana Hospital, Raipur, IND; 6 Spine Surgery, Bhavnagar Institute of Medical Sciences (BIMS), Bhavnagar, IND; 7 Orthopaedics, Sri Madhusudan Sai Institute of Medical Sciences and Research Institute, Chikkaballapur, IND

**Keywords:** gait imbalance, modic changes, pfirrmann grading, schizas grading, upper lumbar disc herniation

## Abstract

Introduction

The upper lumbar spine is anatomically predisposed to neural compression due to a relatively narrow spinal canal, the presence of the conus medullaris and proximal cauda equina, and reduced segmental mobility. This distinct anatomy can lead to a wide range of clinical presentations, from nonspecific polyradiculopathy to cauda equina syndrome. The variability in symptoms and the unique anatomical features often complicate surgical decision-making. The primary objective of this study was to evaluate the association between upper lumbar disc level (L1-L2, L2-L3, L3-L4) and neurological as well as functional presentation. Secondary objectives included correlation with radiological characteristics and demographic variables.

Material and methods

A prospective observational study was conducted at Stavya Spine Hospital and Research Institute over a period of one year. Patients meeting the inclusion criteria underwent a comprehensive clinical evaluation, including a history-taking session, physical examination, and assessment using functional scoring systems, such as the Visual Analogue Scale (VAS) and Oswestry Disability Index (ODI). Patients were asked to map their patterns of back and leg pain, and radiological findings were meticulously documented.

Results

Fifty patients were enrolled consecutively, with an overall mean age of 51.26 ± 14.68 years. Patients with L1-L2 disc herniation were younger (mean age 42.13 ± 11.14 years) compared with those with L3-L4 herniation (54.45 ± 12.24 years). Although patients with L1-L2 disc herniation were younger on average compared with those with L3-L4 herniation, the difference in age across disc levels was not statistically significant. Most L1-L2 disc herniations were classified as Michigan State University (MSU) Type 1B, while L2-L3 and L3-L4 herniations were predominantly MSU Type 2B and Type 3B, respectively. Gait imbalance, extensor plantar response, motor weakness, sensory deficits, reflex changes, and bowel or bladder involvement were more commonly observed in patients with L2-L3 and L3-L4 disc herniations.

Conclusion

Upper lumbar disc herniation demonstrates level-specific demographic and clinico-radiological patterns. L1-L2 herniations occur in relatively younger patients and are predominantly MSU Type 1B, whereas L2-L3 and L3-L4 herniations are more common in older individuals and are typically larger, classified as MSU Type 2B and 3B. Disc herniations at L2-L3 and L3-L4 levels more frequently present with upper motor neuron-like (epiconus-related) neurological features, including gait imbalance, extensor plantar response, motor weakness, sensory deficits, reflex changes, and bowel or bladder involvement. Recognition of these level-dependent clinical patterns is essential for accurate diagnosis and timely surgical decision-making in upper lumbar disc herniation.

## Introduction

The upper lumbar vertebrae (L1-L4) exhibit distinct anatomical characteristics compared to lower lumbar segments, including a narrower spinal canal, the presence of the conus medullaris and origin of the cauda equina, and reduced range of motion. These features render this region more susceptible to neural compression even from a single disc herniation [1]. Clinical presentations are variable, ranging from nonspecific polyradiculopathy to cauda equina syndrome [2]. Classic symptoms of upper lumbar disc herniation (ULDH) include anterior thigh or groin pain [3]. Patients often have a negative straight leg raise test (SLRT) but a positive femoral nerve stretch test (FNST) in 84-94% of cases [4]. Significant motor weakness is uncommon; when present, quadriceps weakness and reduced patellar reflex are most frequently observed [5].

ULDH is relatively rare, accounting for less than 5% of all lumbar disc herniations, with reported incidence ranging from <1% to 10.4% [6]. Variability in definitions contributes to this discrepancy. While some studies define ULDH strictly as L1-L2 and L2-L3 herniations, others include L3-L4 [7]. We included L3-L4 herniations because, although they often resemble lower lumbar pathology anatomically, they can occasionally present with upper lumbar-type clinical or radiological features, particularly in patients with a high conus termination or shortened lumbar segments. Analyses were stratified by level (L1-L2, L2-L3, L3-L4) to differentiate clinical patterns and radiological characteristics across disc levels.

Most patients with ULDH exhibit nonspecific poly-radicular symptoms, with well-localized mono-radiculopathy being rare, contrasting with lower lumbar disc herniations [8-10]. Given these challenges, this study aimed to describe the varied clinical and radiological presentations of upper lumbar disc herniation and characterize the associated dermatomal and myotomal patterns, while highlighting level-specific distinctions between L1-L2, L2-L3, and L3-L4 herniations.

## Materials and methods

A prospective observational study was conducted at our tertiary care center, Stavya Spine Hospital and Research Institute, from October 2022 to September 2023. Approval was obtained from the institutional ethics committee (SSHRI/CS/NS/ProUPID/SK/41/01-22) and the study was registered with the Clinical Trials Registry (CTRI/2022/02/040183). Informed consent was taken. Patients aged 20 to 90 years who presented with low back pain and/or radiculopathy, motor or sensory deficits, bowel or bladder dysfunction, or neurogenic claudication attributable to disc herniation were included. Eligibility required radiological confirmation of single-level disc compression involving any of the first three disc spaces below the conus medullaris. In patients with radiological evidence of multilevel disc disease but clinical features suggestive of single-level acute compression, consensus regarding the symptomatic level was confirmed independently by two investigators. Patients with associated spinal pathologies such as infections, malignancy, or a history of upper lumbar surgery were excluded.

After obtaining written informed consent, all patients underwent a detailed evaluation in the outpatient department. Clinical history included demographic data (age, sex, height, weight), body mass index (BMI), weight change trends, duration and nature of low back pain, side of radiculopathy, presence of gait imbalance, bowel/bladder symptoms, and any visible skin or foot deformities. Pain assessment was done using the Visual Analog Scale (VAS) [11] for back pain, right leg pain, and left leg pain; Oswestry Disability Index (ODI) [12]. Permission to use the Oswestry Disability Index (ODI) was obtained from MAPI Research Trust, the authorized distributor of the instrument. Patients were also asked to chart their pain and radiculopathy distribution on schematic diagrams of the body. The neurological evaluation included assessment of gait; the Romberg sign; nerve tension tests such as the straight leg raise test (SLRT) and femoral nerve stretch test (FNST); muscle wasting; tone; manual motor strength; dermatomal sensory deficits; reflex abnormalities; and, when clinically indicated, a per-rectal examination. Radiological evaluation included: Michigan State University (MSU) classification of disc herniation [13]; Pfirrmann grading of disc degeneration [14]; Pfirrmann grading of nerve root compression [15]; Modic changes in vertebral endplates [16]; and Schizas grading for canal stenosis [17].

Statistical analysis

Statistical analysis was performed using SPSS version 23 (IBM Corp., Armonk, New York, USA). Descriptive statistics were presented as mean ± standard deviation or percentages, as appropriate. The Shapiro-Wilk test was used to assess the normality of distribution. A p-value <0.05 was considered statistically significant. Correlations were evaluated using Spearman's rho (ρ), with correlation coefficients and p-values calculated accordingly. Between-group comparisons were performed using one-way analysis of variance (ANOVA).

## Results

Demographic characteristics by disc level are summarized in Table 1. A significant age difference was observed across disc levels. Symptomatology is summarized in Tables 2, 3.

**Table 1:** Patient demographics and characteristics. Values are presented as mean ± standard deviation or N (%). BMI: body mass index.

Variable	L1–L2 (n = 9)	L2–L3 (n = 18)	L3–L4 (n = 23)	Total (n = 50)	p-value
Age (years), mean ± SD	42.13 ± 11.40	50.75 ± 15.85	54.45 ± 12.24	51.26 ± 14.68	0.895
Gender, n (%)					0.531
Male	6 (66.7)	8 (44.4)	11 (47.8)	25 (50.0)	
Female	3 (33.3)	10 (55.6)	12 (52.2)	25 (50.0)	
BMI (kg/m²), mean ± SD	27.59 ± 10.30	25.99 ± 3.90	25.37 ± 2.90	26.31 ± 3.94	0.848

**Table 2:** Distribution of key neurological and autonomic symptoms by disc level. Data are expressed as N (%).

Variable	Category	L1–L2 (n = 9)	L2–L3 (n = 18)	L3–L4 (n = 23)	Total (n = 50)	p-value
Radiculopathy	Right	0	6 (33.3)	5 (21.7)	11 (22.0)	0.214
	Left	7 (77.7)	7 (38.9)	9 (39.1)	23 (46.0)	
	Bilateral	0	5 (27.8)	9 (39.1)	14 (28.0)	
	None	2 (22.2)	0	0	2 (4.0)	
Imbalance	Present	1 (11.1)	5 (27.8)	4 (17.4)	10 (20.0)	0.432
	Absent	8 (88.9)	12 (66.7)	15 (65.2)	35 (70.0)	
	Unable to stand	0	1 (5.6)	4 (17.4)	5 (10.0)	
Bladder symptoms	Urgency	0	0	1 (4.3)	1 (2.0)	0.588
	Hesitancy	0	0	3 (13.0)	3 (6.0)	
	Retention	0	0	2 (8.7)	2 (4.0)	
	Increased frequency	1 (11.1)	0	1 (4.3)	2 (4.0)	
	Normal	8 (88.9)	18 (100)	16 (69.6)	42 (84.0)	
Bowel symptoms	Constipation	1 (11.1)	1 (5.6)	11 (47.8)	13 (26.0)	0.031

**Table 3:** Comparison of pain and disability scores among disc levels. Values are presented as mean ± standard deviation (range). Between-group comparisons were performed using one-way ANOVA. F-values are reported alongside p-values. A p < 0.05 was considered statistically significant. *Indicates p < 0.05 was considered statistically significant. VAS: visual analogue scale; ODI: Oswestry Disability Index; ANOVA: analysis of variance.

Parameter (mean ± SD, range)	L1–L2 (n = 9)	L2–L3 (n = 18)	L3–L4 (n = 23)	F-value	p-value
VAS (back)	2.7 ± 2.5 (2–10)	2.2 ± 2.0 (2–8)	2.0 ± 3.0 (0–10)	1.59	0.207
VAS (right leg)	0	4.4 ± 3.3 (0–8)	3.9 ± 3.1 (0–9)	6.96	0.002*
VAS (left leg)	4.3 ± 3.4 (0–9)	4.5 ± 2.9 (0–10)	4.9 ± 3.1 (0–9)	0.09	0.914
ODI (%)	55.5 ± 16.7 (26.6–82.2)	56.4 ± 7.9 (48.8–75.5)	65.6 ± 14.7 (26.6–92)	2.59	0.088

Clinical examination findings of the patients are summarized in Tables 4-8. 

**Table 4:** Gait pattern distribution among patients with upper lumbar disc herniation. Data are presented as N (%). Overall comparison performed using the chi-square test. *Indicates p < 0.05 was considered statistically significant.

Type of gait	L1–L2 (n = 9)	L2–L3 (n = 18)	L3–L4 (n = 23)	Total (n = 50)	p-value*
Normal	3 (33.3%)	1 (5.6%)	3 (13.0%)	7 (14%)	
Antalgic	5 (55.6%)	8 (44.4%)	9 (39.1%)	22 (44%)	
Waddling	0	1 (5.6%)	1 (4.3%)	2 (4%)	
Imbalance/myelopathic-like	1 (11.1%)	5 (27.8%)	4 (17.4%)	10 (20%)	
High stepping	0	0	1 (4.3%)	1 (2%)	
Unable to stand	0	3 (16.7%)	5 (21.7%)	8 (16%)	
Overall comparison					0.041

**Table 5:** Presence of Romberg sign. Frequency and percentage of patients showing Romberg sign. Overall comparison performed using the chi-square test. *Indicates p < 0.05 was considered statistically significant.

Romberg sign	L1–L2 (n = 9)	L2–L3 (n = 18)	L3–L4 (n = 23)	Total (n = 50)	p-value*
Present	1 (11.1%)	5 (27.8%)	4 (17.4%)	10 (20%)	
Absent	8 (88.9%)	12 (66.7%)	15 (65.2%)	35 (70%)	
Unable to stand	0	1 (5.6%)	4 (17.4%)	5 (10%)	
Overall comparison					0.048

**Table 6:** Provocative tests: SLR and FNST. B/L: bilateral; SLR: straight leg raise; FNST: femoral nerve stretch test. *Indicates p < 0.05 was considered statistically significant.

SLR result	L1–L2 (n=9)	L2–L3 (n=18)	L3–L4 (n=23)	Total (n=50)	p-value*
Positive (any side)	1 (11.1%)	3 (16.7%)	3 (13.0%)	7 (14%)	
Negative	8 (88.9%)	15 (83.3%)	20 (87.0%)	43 (86%)	
Comparison					0.89
FNST result					
Positive (any side)	3 (33.3%)	5 (27.8%)	8 (34.8%)	16 (32%)	
Negative	6 (66.7%)	13 (72.2%)	15 (65.2%)	34 (68%)	
Comparison					0.67

**Table 7:** Neurological examination findings: motor, sensory, and reflex assessment. Values indicate number (percentage) of patients with deficits in specific myotomes, dermatomes, and reflexes.

Test	Parameters	L1-L2 (9)		L2-L3 (18)		L3-L4 (23)		Total (n = 50)
Motor power	Myotomes/side	Right	Left	Right	Left	Right	Left	-
	L2	0	0	2 (11.1%)	3 (16.6%)	2 (8.7%)	2 (8.7%)	9 (18%)
	L3	0	0	2 (11.1%)	2 (11.1%)	1 (4.3%)	1 (4.3%)	6 (12%)
	L4	0	0	3 (16.6%)	3 (16.6%)	4 (17.4%)	4 (17.4%)	14 (28%)
	L5	0	0	4 (22.2%)	4 (22.2%)	5 (21.7%)	4 (17.4%)	17 (34%)
	S1	0	0	2 (11.1%)	2 (11.1%)	4 (17.4%)	4 (17.4%)	12 (24%)
Sensory examination	Dermatomes/side	Right	Left	Right	Left	Right	Left	-
	L2	0	0	2 (11.1%)	1 (5.5%)	0	1 (4.4%)	4 (8%)
	L3	0	1 (11.1%)	3 (16.6%)	2 (11.1%)	0	2 (8.7%)	8 (16%)
	L4	0	0	4 (22.2%)	1 (5.5%)	1 (4.4%)	1 (4.4%)	7 (14%)
	L5	0	0	5 (27.7%)	2 (11.1%)	3 (13.1%)	1 (4.4%)	11 (22%)
	S1	0	0	2 (11.1%)	2 (11.1%)	3 (13.1%)	1 (4.4%)	8 (16%)
Reflexes	Reflex/side	Right	Left	Right	Left	Right	Left	-
	Adductor	0	0	2 (11.1%)	1 (5.5%)	1 (4.4%)	0	3 (6%)
	Hamstring	0	0	3 (16.6%)	2 (11.1%)	1 (4.4%)	1 (4.4%)	4 (8%)
	Knee	0	1 (11.1%)	6 (33.3%)	3 (16.6%)	6 (26.1%)	4 (17.4%)	12 (24%)
	Ankle	0	0	4 (22.2%)	4 (22.2%)	3 (13.1%)	2 (8.7%)	7 (14%)
	Plantar	0	0	3 (16.6%)	3 (16.6%)	1 (4.4%)	1 (4.4%)	4 (8%)

Motor, sensory, and reflex deficits frequently involved multiple adjacent myotomes and dermatomes, reflecting overlapping neural involvement rather than isolated single-root pathology. Bowel symptoms, particularly constipation, were significantly more frequent in patients with L3-L4 disc herniation (p = 0.031). 

**Table 8:** Anal and perineal neurological findings. Data are shown as N (%).

Test	Parameters	L1-L2 (9)	L2-L3 (18)	L3-L4 (23)	Total (n = 50)
Bulbocavernosus	Present	0	4 (22.22%)	10 (43.47%)	14 (28%)
	Absent	0	0	0	0
	Not tested	9 (100%)	14 (77.77%)	13 (56.52%)	36 (72%)
Anal tone	Present	0	4 (22.22%)	9 (39.13%)	13 (26%)
	Weak	0	0	1 (4.345%)	1 (2%)
	Absent	0	0	5 (21.73%)	5 (10%)
	Not tested	9 (100%)	14 (77.77%)	8 (34.78%)	31 (62%)
Voluntary anal contraction	Present	0	4 (22.22%)	9 (39.13%)	13 (26%)
	Weak	0	0	1 (4.345%)	1 (2%)
	Absent	0	0	5 (21.73%)	5 (10%)
	Not tested	9 (100%)	14 (77.77%)	8 (34.78%)	31 (62%)
Perianal sensation	Normal	9 (100%)	18 (100%)	22 (95.65%)	49 (98%)
	Hypoesthesia	0	0	1 (4.345%)	1 (2%)
	Complete loss of sensation	0	0	0	0

Perianal and bulbocavernosus examinations were performed selectively in patients with clinical suspicion of bowel or bladder involvement, accounting for the high proportion of cases in which these tests were not performed.

Calcification of the disc was present in five patients (10%), including three with L3-L4 herniation and two with L2-L3 herniation. The remaining radiological findings are summarized in Table 9.

**Table 9:** Radiological classification and grading in upper lumbar disc herniation. Data is presented as number N (%). Distribution of cases by MSU classification, Pfirrmann grading of disc degeneration and nerve root compression, Modic changes, and Schizas grading for canal stenosis. ^†^Statistically significant at p < 0.05. *Indicates p < 0.05 was considered statistically significant. MSU: Michigan State University Classification; ODI: Oswestry Disability Index.

Classification	Parameters	L1–L2 (n = 9)	L2–L3 (n = 18)	L3–L4 (n = 23)	Total (n = 50)	p-value*
MSU classification	1A	0	0	3 (13.0%)	3 (6%)	—
	1B	3 (33.3%)	0	2 (8.7%)	5 (10%)	
	1C	0	1 (5.6%)	0	1 (2%)	
	2A	2 (22.2%)	1 (5.6%)	2 (8.7%)	5 (10%)	
	2B	2 (22.2%)	6 (33.3%)	3 (13.0%)	11 (22%)	
	2C	0	0	1 (4.3%)	1 (2%)	
	2AB	1 (11.1%)	1 (5.6%)	0	2 (4%)	
	3A	0	3 (16.7%)	5 (21.7%)	8 (16%)	
	3B	1 (11.1%)	6 (33.3%)	7 (30.4%)	14 (28%)	
Pfirrmann disc degeneration	Grade 2	0	1 (5.6%)	2 (8.7%)	3 (6%)	0.18
	Grade 3	6 (66.7%)	11 (61.1%)	10 (43.5%)	27 (54%)	
	Grade 4	3 (33.3%)	5 (27.8%)	10 (43.5%)	18 (36%)	
	Grade 5	0	1 (5.6%)	1 (4.3%)	2 (4%)	
Pfirrmann nerve root compression	Grade 0	0	0	1 (4.3%)	1 (2%)	0.03†
	Grade 1	3 (33.3%)	3 (16.7%)	4 (17.4%)	10 (20%)	
	Grade 2	4 (44.4%)	4 (22.2%)	3 (13.0%)	11 (22%)	
	Grade 3	2 (22.2%)	11 (61.1%)	15 (65.2%)	28 (56%)	
Modic changes	Type 1	8 (88.9%)	17 (94.4%)	21 (91.3%)	46 (92%)	—
	Type 2	0	0	1 (4.3%)	1 (2%)	
	Type 3	1 (11.1%)	1 (5.6%)	1 (4.3%)	3 (6%)	
Schizas grading	A	2 (22.2%)	0	4 (17.4%)	6 (12%)	0.04†
	B	4 (44.4%)	6 (33.3%)	4 (17.4%)	14 (28%)	
	C	3 (33.3%)	12 (66.7%)	12 (52.2%)	27 (54%)	
	D	0	0	3 (13.0%)	3 (6%)	

Correlation analysis demonstrated a strong positive association between MSU grade and Pfirrmann grading of nerve root compression (ρ = 0.715, p < 0.001). Other clinical and demographic variables showed weak, non-significant correlations with MSU grade.

Multivariate analysis by disc level showed that only VAS for right leg pain (p = 0.002) and pain interference with normal work (p = 0.017) reached statistical significance. Other parameters, including MSU grade (p = 0.085), ODI (p = 0.088), and least pain in the last 24 hours (p = 0.079), were not significant but approached significance. Variables, such as BMI, VAS for back and left leg pain, motor weakness, reflexes, Pfirrmann grades of disc and nerve root compression, and Modic changes, showed no significant associations.

Multivariate analysis against MSU classification showed that diastolic blood pressure (p = 0.046), Pfirrmann grading of nerve root compression (p < 0.001), and pain interference with walking ability (p = 0.041) were statistically significant. Modic changes (p = 0.089) approached significance. Other parameters, including BMI, VAS for back and leg pain, ODI, motor weakness, reflexes, and Pfirrmann grade of disc degeneration, did not reach statistical significance.

## Discussion

ULDH often presents with nonspecific symptoms, including ill-defined polyradiculopathy, muscle weakness, and sensory or reflex changes that are not confined to a single nerve root. This complexity is likely due to the anatomical overlap involving the epiconus, conus medullaris, and cauda equina at the upper lumbar levels, which can all be compressed by a herniated disc. Another contributing factor may be significant cross-innervation among nerve roots in this region [18-20]. In our study, the male-to-female ratio was 1:1, consistent with previous literature reporting similar gender distributions [21,22]. The mean age of patients in the present cohort was 51.3 years, consistent with prior reports suggesting that upper lumbar disc herniation predominantly affects middle-aged to older adults. In contrast, most studies on lower lumbar disc herniation report a younger age distribution [23,24], suggesting that the pathophysiology and degenerative processes in ULDH may differ and be influenced by age-related changes in disc height and facet orientation [25].

L1-L2 and L2-L3 disc herniations predominantly caused left-sided radiculopathy, with pain radiating to the anterior or anterolateral thigh, groin, and anteromedial knee. L3-L4 herniations showed a more balanced side distribution, with pain along the anteromedial thigh and medial leg to the medial malleolus. The slight left-sided predominance observed may be related to anatomical asymmetry or preferential neural compression, though the exact mechanism remains unclear. This variation reflects the complex innervation and overlap in dermatomal distribution at upper lumbar levels [26]. Interestingly, groin pain, a hallmark of L1-L2 herniation, was also observed in L2-L3 herniations in our series, consistent with other studies that report groin pain in nearly 38% of L2-L3 cases [27], likely due to overlapping innervation by L1, L2, and L3 roots. Although obesity has been implicated in lower lumbar degenerative disc disease, our analysis did not demonstrate a significant association between BMI and disc size or clinical severity in upper lumbar disc herniations, indicating a potentially different biomechanical or pathophysiological profile at these levels.

An et al. (2021) demonstrated that lower-level lumbar disc herniations, traditionally not associated with groin pain, can indeed produce discogenic groin symptoms through overlapping innervation involving the L1-L2 spinal ganglia and nociceptive pathways. Their study highlighted that even herniations at lower lumbar levels may trigger referred groin pain, challenging the classical view that groin pain is restricted to upper lumbar disc disease. Clinically, patients presenting with discogenic groin pain showed significant improvement following percutaneous endoscopic discectomy, supporting the efficacy of targeted surgical intervention. These findings emphasize the importance of recognizing atypical pain patterns in lumbar disc disease and considering anatomical neural overlap when planning management [28,29]. Conus medullaris syndrome-like presentations, such as urinary urgency, frequency, or hesitancy, were observed in six out of 50 patients (12%). Bladder symptoms were observed exclusively in patients with L3-L4 disc herniation, predominantly in the form of urgency, hesitancy, and retention. Despite these complaints, none demonstrated urodynamic abnormalities or required catheterization, suggesting early or incomplete epiconus involvement rather than established conus medullaris syndrome.

Neurological deficits were common, most frequently affecting the quadriceps, followed by hip flexors and ankle dorsiflexors. Patellar reflexes were often reduced, consistent with L3-L4 involvement. Multilevel weakness across L2-L4 myotomes was frequently observed, making precise localization difficult. This pattern likely results from overlapping motor innervation and possible epiconus involvement [30]. Sensory disturbances were primarily reported in the anterior thigh and medial leg. In patients with L1-L2 herniation, sensory loss extended to the inguinal region and suprapubic area. Loss of abdominal reflexes was noted in six patients (12%), all of whom had high-grade L1-L2 herniations. These patients exhibited dermatomal sensory loss from L1 to L4 and proximal myotomal weakness, resembling upper motor neuron signs despite normal reflexes and absent clonus, potentially due to epiconus compression [31]. The SLR test was positive in a few patients, while the FNST was frequently positive, especially in L1-L2 and L2-L3 herniations, with a decline at L3-L4, indicating its utility for upper lumbar radiculopathy [32].

Preoperative VAS was 7.3 ± 1.7, with leg pain scores of 3.58 ± 2.4 (right) and 3.62 ± 2.3 (left). Mean ODI was 60.6 ± 12.2, highest in L3-L4 (65.6) and lowest in L1-L2 (55.5). Analgesic response was greatest in L1-L2 (6.03) and least in L3-L4 (4.5). These findings suggest that patients with lower-level upper lumbar herniations experienced greater disability and poorer analgesic response, potentially due to more extensive neural involvement and increased MSU disc grades [33,34].

Radiologically, L1-L2 herniations were generally smaller, with most (eight of nine) being MSU grade 1 or 2. In contrast, L3-L4 herniations were larger, with 17 of 21 classified as grade 2 or 3. Grade 3 herniations, frequently associated with thecal sac effacement, were seen in eight patients, six of whom had L3-L4 involvement. All grade 3 herniations caused nerve root compression (grades 2-3), and consistent with previous radiological findings of moderate to severe canal stenosis (Schizas grade C or D) [35,36]. In contrast, all grade 3 herniations showed moderate to severe compression, reinforcing the value of MSU classification in predicting nerve root involvement [37].

Disc degeneration, assessed by Pfirrmann grading, was most severe in L3-L4 herniations, with 71.4% graded as Pfirrmann 4 or 5. In contrast, 77.8% of L1-L2 herniations were graded as Pfirrmann 2 or 3. Modic changes were present in 30% of patients overall, with Modic type 2 being the most common. L3-L4 herniations had the highest prevalence of Modic changes, followed by L2-L3 and L1-L2 [38]. Facet joint hypertrophy and ligamentum flavum thickening, most frequently observed at L3-L4, contribute to canal stenosis and symptom severity, as reported in previous studies [39,40].

MSU disc grade correlated strongly with nerve root compression (ρ = 0.715, p < 0.001) and weakly with age, ODI, right leg pain, and disc degeneration, but not with sex, BMI, left leg pain, FNST, or SLR. Modic changes were not associated with pain or disability. This study highlights the complexity of ULDH, where clinical presentation does not always correlate precisely with radiological findings due to overlapping neural structures. MSU disc grading correlated well with root compression severity but not strongly with clinical scores, reinforcing the need for comprehensive evaluation. FNST was more sensitive than SLR in detecting upper lumbar radiculopathy, particularly in L1-L2 and L2-L3 herniations. Patients with L3-L4 herniations had more severe disc disease and worse functional outcomes [41].

This study has several limitations. The relatively small sample size may limit the generalizability of findings. Additionally, we did not evaluate post-treatment outcomes, precluding correlation between preoperative findings and surgical results. Further studies with larger cohorts and postoperative follow-up are warranted to assess the prognostic value of clinical and radiological parameters in ULDH.

## Conclusions

Upper lumbar disc herniation demonstrates clear level-dependent clinical and radiological patterns. L1-L2 herniations tend to occur in relatively younger patients and are generally smaller in size, whereas L2-L3 and L3-L4 herniations are more frequent in older individuals and are associated with larger disc size, higher MSU grades, and more severe neurological involvement. BMI did not demonstrate a significant association with disc size or clinical severity, suggesting that factors other than body habitus may play a more prominent role in upper lumbar disc pathology. Lower upper lumbar levels more commonly exhibit gait imbalance, positive Romberg sign, extensor plantar response, and bowel or bladder symptoms, reflecting epiconus-related neural compromise. Recognition of these level-specific presentations is essential for accurate diagnosis and optimal clinical decision-making in ULDH.
